# Sugarcane Light-Colored Lignin: A Renewable Resource for Sustainable Beauty

**DOI:** 10.3390/ijms242115941

**Published:** 2023-11-03

**Authors:** Inês F. Mota, Filipa Antunes, Joana F. Fangueiro, Carina A. E. Costa, Alírio E. Rodrigues, Manuela E. Pintado, Patrícia S. Costa

**Affiliations:** 1 CBQF—Centro de Biotecnologia e Química Fina—Laboratório Associado, Escola Superior de Biotecnologia, Universidade Católica Portuguesa, Rua Diogo Botelho 1327, 4169-005 Porto, Portugal; inesmota@gmail.com (I.F.M.); fantunes@ucp.pt (F.A.); jfangueiro@ucp.pt (J.F.F.); mpintado@ucp.pt (M.E.P.); 2 Amyris Bio Products Portugal Unipessoal Lda, Rua Diogo Botelho 1327, 4169-005 Porto, Portugal; 3 LSRE-LCM—Laboratory of Separation and Reaction Engineering—Laboratory of Catalysis and Materials, Faculty of Engineering, University of Porto, Rua Dr. Roberto Frias, 4200-465 Porto, Portugal; arodrig@fe.up.pt; 4 ALiCE—Associate Laboratory in Chemical Engineering, Faculty of Engineering, University of Porto, Rua Dr. Roberto Frias, 4200-465 Porto, Portugal

**Keywords:** light-colored lignin, cosmetics, sugarcane bagasse

## Abstract

Lignin has emerged as a promising eco-friendly multifunctional ingredient for cosmetic applications, due to its ability to protect against ultraviolet radiation and its antioxidant and antimicrobial properties. However, its typical dark color and low water solubility limit its application in cosmetics. This study presents a simple process for obtaining light-colored lignin (LCLig) from sugarcane bagasse (SCB) alkaline black liquor, involving an oxidation treatment with hydrogen peroxide, followed by precipitation with sulfuric acid. The physico-chemical characterization, antioxidant and emulsifying potential of LCLig, and determination of its safety and stability in an oil-in-water emulsion were performed. A high-purity lignin (81.6%) with improved water solubility was obtained, as a result of the balance between the total aromatic phenolic units and the carboxylic acids. In addition, the antioxidant and emulsifying capacities of the obtained LCLig were demonstrated. The color reduction treatment did not compromise the safety of lignin for topical cosmetic applications. The emulsion was stable in terms of organoleptic properties (color, pH, and viscosity) and antioxidant activity over 3 months at 4, 25, and 40 °C.

## 1. Introduction

The demand for natural and sustainable ingredients in personal care products is growing globally due to the increasing concerns about the safety and environmental impact of many synthetic ingredients used in the cosmetic industry. Additionally, evolving consumer behavior and lifestyles are creating opportunities for natural and eco-friendly ingredients. In this context, lignin has gained special prominence as a natural, renewable, biodegradable, and non-toxic cosmetic ingredient [1,2,3]. Research in this field has experienced significant growth in recent years, with numerous studies demonstrating the tremendous potential of lignin as a natural photo-protector, antioxidant, antimicrobial, and skin-whitening agent [3,4,5,6]. Robust scientific evidence supporting the safety of lignin has further fueled the interest of both academic and industrial researchers. Lignin has been shown to be non-irritating to the eyes and skin, as well as non-mutagenic and non-genotoxic [3,4,5]. Despite the proven potential of lignin, its inherent dark brown color poses a challenge in color-dependent applications, thus hindering its valorization [7]. Although lignin is nearly colorless in its natural state, the processing of biomass can generate various chromophores. The nature of these chromophores depends on different factors, such as the biomass source and extraction conditions. However, due to the structural complexity and variability of lignin, the exact mechanism of chromophore formation is not fully understood [8]. 

Efforts to remove or reduce chromophores from the lignin structure date back to the 1980s. Previous studies have explored several methods, including chemical blocking of free phenolic hydroxyl groups to effectively reduce the color of lignin [9,10], and some have combined this with oxidation with chlorine dioxide, oxygen, hydrogen peroxide [11,12], or whitening agents (e.g., sodium borohydride) [13]. Alternatively, recent studies have focused on the removal or suppression of chromophoric structures through fractionation with solvents (e.g., methanol/water mixtures) or precipitation at different pH values [7,14,15,16], oxidation by UV radiation [17], biological color reduction by fungi [18], and modification of the chemical structure, morphology, or size of lignin (e.g., acetylation, alkylation, ball milling, drying method) [7,19,20,21,22]. It has been suggested that mild delignification, lignin re-slurring in acidic water, and ball milling can result in lighter lignins [7]. Zhang and co-workers have shown that methanol/water fractionation results in lighter lignins due to the removal of condensed and unsaturated structures [16]. Lignin oxidation with UV radiation was proposed by Wang and co-workers, where lignin was dissolved in tetrahydrofuran and irradiated with UV light for 200 h to protoxidize the phenoxyl groups into colorless aliphatic acid [17]. While substantial efforts have been made, there remains a need to identify simpler, cost-effective, and environmentally friendly processes for reducing the color of lignin. Hydrogen peroxide, a well-known oxidant, is often used in lignin depolymerization experiments or studies involving pulp bleaching, either alone or in combination with other oxidants [23,24,25]. Hydrogen peroxide is a non-toxic chemical that readily decomposes into molecular oxygen and water. In an acidic medium, hydrogen peroxide acts as a strong oxidant. However, in an alkaline medium, it reacts mainly as a nucleophile with carbonyl and conjugated carbonyl structures (e.g., quinoid structures), effectively removing chromophore structures without degrading the lignin structural network [26]. Recent studies have achieved success in obtaining transparent wood through mild alkaline treatments combined with hydrogen peroxide concentrations of up to 25 wt% [27,28,29]. Li and co-workers have demonstrated that over 80% of the bulk lignin can be preserved in transparent wood material obtained through a combined alkaline/hydrogen peroxide treatment of up to 3 wt% [30]. Building upon these findings, this study proposes, for the first time, the use of mild hydrogen peroxide treatment in alkaline liquor to reduce the color of lignin extracted from SCB. The resulting light-colored lignin (referred to as LCLig) was characterized in terms of composition, color, particle size, molecular weight, and functional groups. Its antioxidant and emulsifying properties were also investigated. Furthermore, the safety and stability of LCLig were evaluated in an oil-in-water (o/w) emulsion formulation.

## 2. Results and Discussion

### 2.1. Physico-Chemical, Structural, and Thermal Analyses

Analysis of Table 1 reveals a substantial reduction in the color of LCLig under the applied process conditions. The purity and carbohydrate and ash contents of LCLig were 81.6 ± 3.6%, 3.50 ± 0.40%, and 6.03 ± 0.01%, respectively (Table 1). The color of LCLig was evaluated quantitatively using the International Commission on Illumination CIE L*a*b* color space, showing a lightness value of 73, indicating a light color. The color coordinates a* and b* values were 2.5 and 24.5, respectively (Table 1). Comparing the results with those reported by Antunes and co-workers [31] for a lignin obtained from SCB (L*60/a*6/b*19), it can be concluded that the proposed simple process successfully reduced the color of lignin extracted from SCB.

The ^31^P NMR analysis allowed the quantification of the syringyl (S), guaiacyl (G), and *p*-hydroxyphenyl (H) free phenolic groups, as well as carboxylic, aliphatic, and condensed structures containing free phenolic groups [32] (Table 2). The content of total phenolic units of LCLig was 0.89 mmol/g _lignin_, which is lower than that of the original SCB lignin, i.e., without H_2_O_2_ treatment (2.31 mmol/g _lignin_) [31]. Free phenolic hydroxyl groups are one of the most important structural features regarding the reactivity and antioxidant properties of lignin [31,33]. Therefore, the content of phenolic hydroxyl groups is usually associated with the dark brown color of lignin. Thus, reducing its color can cause a decrease in antioxidant activity. The TPC variations might be attributed to condensation, re-polymerization, or char formation processes that may occur during the color reduction treatment [34,35]. The carboxylic acids content of LCLig was 1.76 mmol/g _lignin_, again a lower value than that of the original lignin (0.74 mmol/g _lignin_) [31]. 

**Figure 1:**
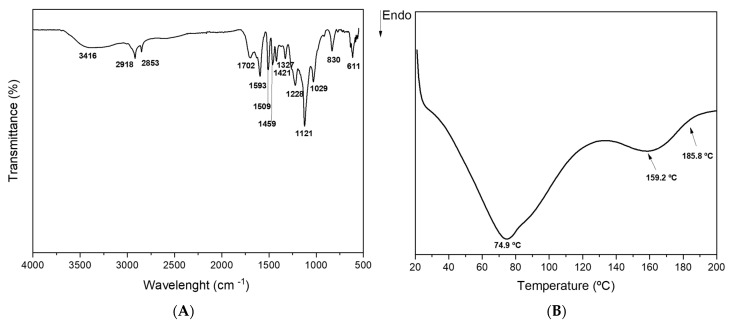
(**A**) FTIR spectrum and (**B**) DSC thermogram of light-colored lignin (LCLig).

According to the ATR-FTIR spectrum (Figure 1A), and in comparison to our previous findings [31,35], the lignin structure was not significantly affected by the removal of some chromophores. This result was expected since, in alkaline medium, hydroxide peroxide reacts with both aliphatic and aromatic structures of lignin, thus removing some chromophore structures without compromising the lignin structure network [26]. Nevertheless, the FTIR spectrum (Figure 1A) revealed a pronounced band between 3650 and 3050 cm^−1^ attributed to hydroxyl groups in phenolic and aliphatic structures [36], probably due to lignin depolymerization triggering condensation reactions [23,37]. Some peaks were also observed in the carbonyl/carboxyl region, 1700–1680 cm^−1^, corresponding to unconjugated C=O stretching (vibrations of unconjugated ketones, esters, or carboxylic acids) [38]. The region between 3000 cm^−1^ and 2830 cm^−1^, where bands at 2918 cm^−1^ and 2853 cm^−1^ were detected, corresponds to C-H stretching in methyl, methylene, and methoxyl groups [39]. Aromatic skeletal vibrations were assigned to the bands at 1593 cm^−1^ (characteristic of condensed G-units), 1509 cm^−1^, 1459 cm^−1^, and 1421 cm^−1^ [39]. The band at 1593 cm^−1^ corresponded to the aromatic skeletal vibration characteristic of condensed guaiacyl (G) units, and the band at 1327 cm^−1^ can be related to aryl ring breathing with C-O stretch characteristic for the syringyl (S) ring plus G ring condensed, while the band at 1228 cm^−1^ can be attributed to G units (condensed > etherified G units, not a relevant band). The band at 830 cm^−1^ was assigned to C-H out-of-plane bending in positions 2, 5, and 6 of S units and all positions of *p*-hydroxyphenyl (H) units [33,40]. The presence of *p*-coumaric esters (typical for GSH lignins) is shown in both samples at 1121 cm^−1^ and 1029 cm^−1^, indicating the C-O stretching in C-O-C linkages between S and G units [33,40,41]. There was a remarkable band at 611 cm^−1^ (Figure 1A) attributed to the C-H bending vibration of aromatic ring substituents, specifically, the C-H out-of-plane bending of meta-substituted aromatic rings in lignin [42].

From Figure 1B, it can be observed that LCLig is stable up to 200 °C, which is a relevant property for cosmetic and other industrial applications. Previous studies have already reported the thermostability of lignins at temperatures up to 200 °C, with dehydration and decarboxylation processes being the most common events observed within this temperature range [35,43,44,45]. Nevertheless, the thermostability of lignin is affected by its degree of polymerization and branching, and the presence of functional groups such as hydroxyl, methoxyl, and carboxyl groups [43]. According to the DSC results (Figure 1B and Table 3), the profile of LCLig presented two main endothermic events. The first event, at 74.9 °C, was caused by water removal (dehydration), as confirmed in the literature [43]. LCLig showed an enthalpy of −65.76 J/g and a peak height of −0.6585 mW/mg (Table 3). The second event, at 159.2 °C, described a different thermal profile from 140 °C. These peaks are related to decarboxylation reactions involved in the removal of carboxyl groups from the lignin structure. Based on the NMR analysis (Table 2), LCLig is rich in carboxyl and aliphatic hydroxyl groups and, therefore, this is an expected peak (−8.367 mW/mg) (Table 3).

### 2.2. Particle Size and Molecular Weight

The particle size distribution, weight-average (Mw) and number-average (Mn) molecular weights, and polydispersity index (Mw/Mn) are shown in Table 4. The volume-based mean diameter (D[4,5]) and the surface-area-based mean diameter (D[3,4]) of the LCLig were 13.88 μm and 6.00 μm, respectively. The cumulative particle size at 10%, 50%, and 90%, represented as Dv(10), Dv(50), and Dv(90), of the LCLig were 2.83 μm, 8.07 μm, and 27.24 μm, respectively. Some previous studies reported a reduction in the molecular weight of lignin after bleaching due to its oxidative cleavage [23,46,47]. The Mw, Mn, and polydispersity index of the original SCB lignin were 15,199 Da, 10,793 Da, and 1.41, respectively. A possible explanation for these differences is that the mild conditions applied in the present study, such as low extraction temperatures and concentration of H_2_O_2_, may have prevented extensive fractionation or degradation of the lignin, resulting in minimal changes in its molecular weight. This suggests that the use of mild treatment conditions can be beneficial in maintaining the structural integrity and properties of lignin, while still achieving the desired modifications [48].

### 2.3. Solubility

**Figure 2:**
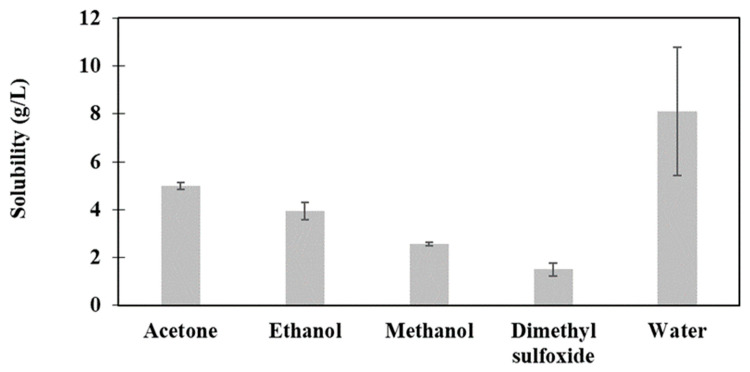
Solubility of the light-colored lignin (LCLig) in different solvents.

The data from Figure 2 indicate that the color reduction process induced a suitable water solubility in LCLig and comparable lower solubility in the remaining solvents tested. The treatment may break down certain chemical structures within the lignin that hinder its solubility in water. These variations are influenced by its ability to establish intermolecular interactions with aliphatic, carbonyl, and hydroxyl groups present in the lignin structure [49]. Notably, the NMR analysis confirmed that LCLig has a relatively suitable concentration of phenolic hydroxyl groups that can contribute to this water solubility (Table 2) [48].

### 2.4. Total Phenolic Compounds (TPC) and Antioxidant Potential

The TPC and antioxidant properties of LCLig are summarized in Table 5. LCLig presented antioxidant activity assessed by both methods. When comparing the values with those reported in the literature for the original SCB lignin (330.3 ± 19.1 mg GAE/g for TPC, 5840.9 ± 130.9 µmol TE/for ORAC, and an IC_50_ of 0.2 ± 0.1 mg/mL for ABTS), a slight decrease in the TPC and antioxidant activity was observed [35]. This decrease appears to be related, at least in part, to the aliphatic hydroxyl groups, which are known to have a negative contribution to radical scavenging activity [5,50]. These findings are consistent with previous research on the modification of SCB lignins through oxidation, acetylation, or epoxidation [38,51].

### 2.5. Emulsion Stability Index (ESI)

The emulsifying potential of lignin in an o/w emulsion was investigated, and the results are depicted in Table 6. According to the results, an LCLig concentration-dependent effect was observed, with the *ESI* increasing from approximately 58 to 90% as the concentration of LCLig in the emulsion increased from 1.0 to 7.5 wt%. LCLig demonstrated effective stabilization of emulsions containing a mixture of fatty alcohols at a concentration of 2.5 g per g of oil phase. While the precise mechanism of lignin self-assembly and colloidal behavior in emulsions remains unclear [52], studies have suggested that lignin stabilizes emulsions through adsorption at the o/w interface, preventing droplet coalescence by electrostatic and steric repulsion [53]. As per the findings of this study, a relatively high concentration of LCLig (ranging from 1 to 10 wt%) is required compared with typical emulsifiers to effectively reduce the surface tension and solvate the oil droplets. Specifically, for a mixture of fatty alcohols such as cetyl stearyl alcohol—commonly used in cosmetic products—a concentration of 10 wt% of LCLig was found to be effective in reducing surface tension and stabilizing the emulsions (Table 6). Indeed, the high *ESI* of LCLig even at lower concentrations (1.0 wt%), suggests its potential as a co-emulsifier candidate in cosmetic formulations, potentially reducing the overall concentration of synthetic emulsifiers in cosmetic products.

### 2.6. Cytotoxicity, Mutagenicity, and Skin Sensitization

**Figure 3:**
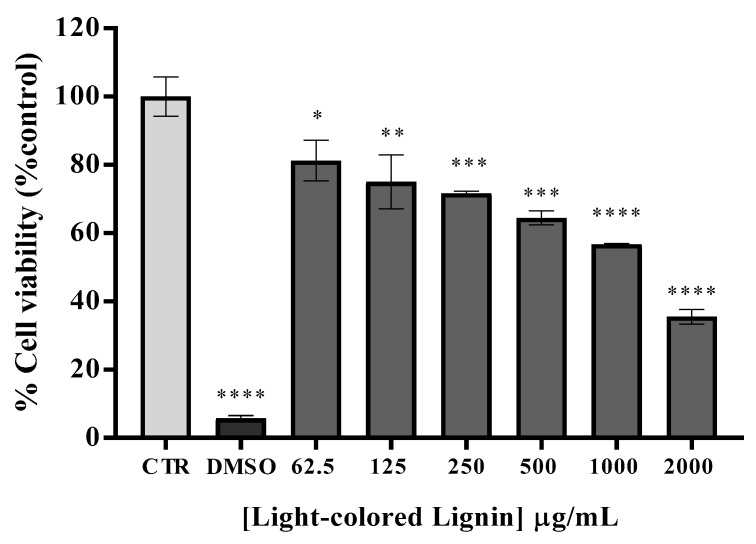
Safety assessment in HaCAT line cell viability after 24 h of incubation with serial dilutions of light-colored lignin. Results are expressed as % of PrestoBlue reduction vs control (CTR, DMEM). DMSO (10%) represents the positive control. Results are expressed as the mean ± SD performed in triplicate. All the results are expressed as the mean ± SD of two independent assays, performed in quadruplicate. * *p* < 0.05, ** *p* < 0.01, *** *p* < 0.001, **** *p* < 0.0001 (ANOVA, Tukey HSD); a* *p* < 0.05—different from non-treated cells (CTR).

The effect of LCLig on HaCAT cell viability was evaluated by PrestoBlue fluorescence assay with six different concentrations (61.5, 125, 250, 500, 1000, and 2000 μg/mL) for 24 h. As demonstrated in Figure 3, all the tested concentrations were statistically different from the control. The sample LCLig was not cytotoxic up to 250 µg/mL. The results revealed a dose–response effect, where higher concentrations of LCLig resulted in lower cell viability. The maximum tested concentration of LCLig (2000 μg/mL) caused a significant decrease in cell viability to a value of 35.5% (*p* ˂ 0.0001).

**Figure 4:**
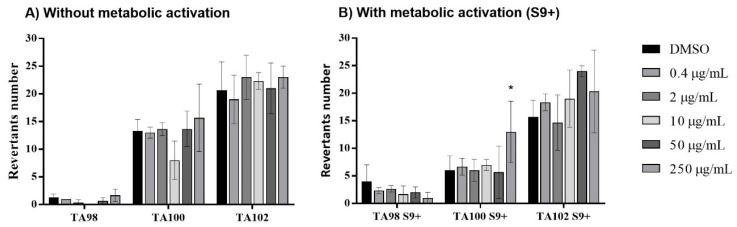
Number of revertant *Salmonella typhimurium* individuals after exposure to different concentrations of the LCLig, (**A**) without metabolic activation and (**B**) with metabolic activation (S9+). Results are presented for the three mutant strains tested, TA98, 100, and 102. All the results are expressed as the mean ± SD of two independent assays, performed in quadruplicate. * *p* < 0.05, (ANOVA, Tukey HSD) different from the control (DMSO).

Regarding the mutagenicity of the LCLig in *S. typhimurium* in the three tested strains with (S9+) (Figure 4B) and without metabolic activity (Figure 4A), all the tested concentrations (0.4 to 250 µg/mL) revealed no significant reversion (*p* < 0.05). Conversely, concerning the strain TA100 without metabolic activation (Figure 4A), only a concentration of 250 µg/mL revealed a statistically significant increase in mutants relative to the control (*p* < 0.05). In this respect, these results revealed that LCLig did not induce mutagenicity in all tested strains up to 250 µg/mL, being this maximum concentration more sensitive to base-pair substitution mutations.

The skin sensitization potential was assessed through the Direct Peptide Reactivity Assay (DPRA), which quantifies the reactivity of chemicals towards synthetic peptides. The DPRA method contributes to the assessment of the skin sensitization potential of chemicals and refers to the molecular initiating event of the adverse outcome pathway, i.e., the covalent interaction with proteins [54]. Based on the results presented in Table 7, LCLig was not observed to be a sensitizer up to the tested maximum concentration (1.4 mg/mL).

### 2.7. Accelerated Stability

The stability of an o/w emulsion containing 5 wt% LCLig was evaluated according to the ISO 22716:2007 guidelines for the cosmetic industry [55]. The initial color coordinates L*/a*/b* were 53/5.7/20.5. The stability tests were conducted on both the blank and 5 wt% LCLig o/w emulsion (Table 8). The control was set at 4 °C (formulation that must remain unchanged during the whole life cycle of the product), and samples were maintained at 25 °C (set as shelf sample) and 40 °C (accelerated stability). The variation in several parameters is depicted in Table 8. Regarding the physical appearance of both emulsions (blank and 5 wt% LCLig o/w), good physical stability was observed during the testing period for all temperatures (4, 25, and 40 °C). No phase separation, creaming, flocculation, or sedimentation was observed, indicating the good physical stability of the emulsions. Both emulsions maintained their homogeneity and smooth texture, as well as good spreadability, throughout the stability testing period. The color of the 5 wt% LCLig o/w emulsion was stable under all the tested conditions, as per the results presented in Table 8. The pH of the o/w emulsion containing 5 wt% LCLig revealed more consistent values compared with the blank o/w emulsion, indicating that LCLig was able to stabilize the pH. Maintaining an optimal pH range of 4.5–6.5 in cosmetic formulations is desirable as it helps to protect the *stratum corneum* (the outermost layer of the skin) and stabilizes the hydrolipidic film. Another notable finding was that the addition of LCLig did not affect the rheological properties of the final product—concretely, the viscosity. The antioxidant activity of the 5 wt% LCLig o/w emulsion remained stable after a 3-month period, and both formulations showed no microbial contamination. This confirms the safety of the emulsion for topical application and good manufacturing practices.

## 3. Materials and Methods

### 3.1. Materials

Hydrogen peroxide (H_2_O_2_, 35%) was purchased from Labchem, Zelienople, PA, USA. Sulfuric acid (H_2_SO_4_, 95–98%), acetone (>99.8%), dimethylformamide (DMF, 99.8%), anhydrous pyridine (99.8%), deuterated chloroform (CDCl_3_, 99.8%), cholesterol, chromium (III) acetyl-acetonate, (phosphitylating reagent (2-chloro-4,4,5,5-tetramethyl-1,3,2-dioxaphospholane), dimethyl sulfoxide (DMSO, >99.9%), Folin–Ciocalteu phenol reagent (2 M), 2,2′-Azino-bis-(3-ethylbenzthiazoline-6-sulfonic acid) diammonium salt (ABTS, ≥98%), potassium persulfate (≥99.0%), (±)-6-Hydroxy-2,5,7,8-tetramethylchromane-2-carboxylic acid (Trolox, 97%), butylated hydroxytoluene (BHT, ≥99%), [2,2′-azobis(2-amidi-nopropane) dihydrochloride] (AAPH), acetonitrile (≥99.9%); l-lysine, cinnamic aldehyde, cysteine, trifluoroacetic acid and gallic acid, and sodium carbonate were purchased from Sigma-Aldrich, St. Louis, MO, USA. Ethanol (>99.8%) was obtained from Honeywell. Methanol was purchased from VWR Chemicals (>99.8%). Dulbecco’s Modified Eagle Medium (DMEM, GlutaMAX™, Thermo Fischer Scientific, Waltham, CA, USA), TrypLEX express enzyme, phosphate buffer, fetal bovine serum, penicillin–streptomycin (pen-strep), and dimethyl sulfoxide (DMSO) were obtained from Gibco, Thermo Fischer Scientific, Waltham, CA, USA. Human keratinocyte cell line (HaCAT) and PrestoBlue cell viability reagent Fluorescein [3′,6′-dihydroxyspiro (isobenzofuran-1 [3H], 9′ [9H]-xanten)-3-one] were obtained from Thermo Fisher Scientific, Waltham, CA, USA. Salmonella typhimurium containing a deletion mutation for histidine (His), with the following mutations: the type frameshift (TA98), base-pair substitution (TA100) and reversion/transversion (TA102), 2-Aminoantracene, and ampicillin were purchased from Moltox, VWR, Carnaxide, Portugal. Nutrient broth-2 and phosphate buffer were purchased from Oxoid, Thermo Fischer Scientific, Waltham, CA, USA. Liver homogenate, S9 (Aroclor 1254-induced Sprague Dawley rat liver) was obtained from Xenometrix, Thermo Fischer Scientific, Waltham, CA, USA. Glycerin, lanette, and refined shea butter were supplied by Acofarma, Águeda, Portugal. Solagum AX was acquired from Seppic, Courbevoie, France. Tegocare PBS 6MB was supplied by Evonik, Essen, Germany and squalene was acquired from Amyris, Emeryville, CA, USA. Euxyl PE 9010 was acquired from LotionCrafter, Washington, DC, USA. Lanette^®^ O (cetyl stearyl alcohol) was acquired from BASF, Ludwigshafen, Germany.

### 3.2. Lignin Extraction and Color Reduction Process

The SCB was air-dried overnight in a convection oven at 40 °C. Then, the process of SCB delignification was performed with 2 wt% sodium hydroxide solution (Labchem, Zelienople, PA, USA) at 90 °C for 0.5 h with a liquid–solid ratio of 15 using a high-pressure reactor (Parr Instruments company, model 4551, Moline, IL, USA). After lignin extraction, the black liquor was separated from the solid and treated with hydrogen peroxide 35% prior to lignin precipitation. In a preliminary study, different loads of oxidant and sulfuric acid were tested, and based on the results, 9% (*v*/*v*) of H_2_O_2_ and 2.5% (*v*/*v*) of H_2_SO_4_ were identified as the optimal process conditions and applied to the color reduction treatment. The reaction was conducted at 85 °C (water bath, Julabo SW22, Julabo, Seelbach, Germany) for 1.5 h. Afterwards, lignin was precipitated with sulfuric acid, and thoroughly washed with tap water by means of vacuum filtration using a paper filter (Whatman 113). The LCLig cake was spray-dried (Büchi Mini Spray Dryer model B-290) using a water suspension at 3 wt% and the following operating conditions: 65% aspirator rate (equivalent to about 27.5 m^3^/h), flow height 40–45 mm (equivalent to 667–831 L/h), pump speed 12% (equivalent to 4 mL/min), and inlet temperature 160 °C. These operating conditions resulted in an outlet air temperature of approximately 85 °C.

### 3.3. Moisture, Ashes, Total Lignin, and Carbohydrates Content

The moisture and inorganic content of the lignin samples were determined gravimetrically at 105 °C (Venti-Line Model, VWR, Carnaxide, Portugal) and 550 °C (Nabertherm, Lilienthal, Germany), respectively, until constant weight. The crucibles were previously calcined overnight at 550 °C. Total carbohydrates and soluble and insoluble lignin were determined after acid hydrolysis as described by Ajao and co-workers [7] using a high-performance liquid chromatograph equipped with a refractive index detector (HPLC-RI) and Aminex HPX 87H column 300 × 7.8 mm (Bio-rad laboratories, Algés, Portugal). The chromatograms were run in isocratic mode at 0.6 mL/min and 50 °C. The mobile phase employed was 5 mM sulfuric acid solution and the injection volume was 10 µL. All samples were analyzed at least in duplicate.

### 3.4. Color

The color measurements were performed according to the Commission Internationale de l’Eclairage CIELAB color scale over a black background on a portable reflection spectrophotometer (CR-410 Chroma Meter, Konica Minolta, Porto, Portugal) with a specular-component-excluded geometry. For blank calibration, the equipment was calibrated with a CR A44 Calibration Plate. The values of L* (lightness and darkness), a* (red and green), and b* (yellow and blue) were obtained.

### 3.5. Structural Characterization

#### 3.5.1. Attenuated Total Reflectance—Fourier-Transform Infrared Spectroscopy (ATR-FTIR)

The lignin samples were analyzed by the ATR-FTIR (Perkin Elmer Frontier) by direct transmittance in a single-reflection ATR System. The equipment was configured for 16 scans in a range of 4000–550 cm^−1^ and a resolution of 4 cm^−1^. For all spectral manipulation, Perkin Elmer FTIR Software Spectrum 10.1.0 (Perkin Elmer, Waltham, MA, USA) was used.

#### 3.5.2. ^31^P Nuclear Magnetic Resonance (NMR)

The quantitative ^31^P NMR analysis was performed as described by Costa and co-workers [32]. Briefly, approximately 40 mg of each lignin was weighed and dissolved in 0.4 mL of anhydrous pyridine and deuterated chloroform (1.6:1.0, *v*/*v*). The mixture was left at room temperature overnight with continuous stirring. Cholesterol (200 μL at 19 mg/mL, internal standard) and 50 μL of chromium (III) acetyl-acetonate (11.4 mg/mL, relaxation agent) were added and allowed to react for 2 h. Phosphitylating reagent (2-chloro-4,4,5,5-tetramethyl-1,3,2-dioxaphospholane, 100 μL) was added and the mixture was transferred to a 5 mm OD NMR tube. The phosphitylated samples were analyzed by ^31^P NMR spectroscopy using a Bruker AVANCE III 400 spectrometer operating at 400 MHz, at 298 K. The spectrum was acquired across 30 min with 10 s relaxation time, 45° pulse angle, and 4 s pulse delay.

### 3.6. Differential Scanning Calorimetry (DSC)

The glass transition temperature of LCLig was assessed using differential scanning calorimetry as described by Fangueiro and co-workers [56]. The sample was weighed (5 g) into an aluminum pan and analyzed under nitrogen atmosphere at a heating rate of 10 °C/min from 30 to 210 °C (Netzsch-Gerätebau GmbH DSC 204 F1 Phoenix^®,^ Selb, Germany). Purging with nitrogen gas at a flow rate of 40 mL/min was performed to maintain an inert atmosphere. 

### 3.7. Particle Size 

The particle size of lignin was measured using a MasterSizer Hydro 3000 equipped with a Hydro EV dispersion unit (Malvern Instruments, Worcestershire, UK), utilizing an obscuration range of 5–10%. The sample was dispersed in water and subjected to external ultrasonication for 5 min. Prior to analysis, the sample was submitted to 1 min of ultrasound treatment and stirred at 2500 rpm. For the particle size distribution, spherical particles with a refractive index of 1.64 and an absorption index of 0.01 were considered. 

### 3.8. Gel Permeation Chromatography (GPC)

The number-average (Mn), weight-average (Mw), and polydispersity (PD) of the LCLlig were obtained as described by Mota and co-workers [57] with slight modifications. An Agilent 1260 Infinity II system equipped with a UV detector and quaternary pump was used. An Agilent gel Oligopore column 300 × 7.5 mm with a nominal particle size of 6 µm was used in series with an Agilent gel Mesopore column 300 × 7.5 mm with nominal particle size of 3 µm, measuring molecular weights up to 4500 g/mol and 25,000 g/mol, respectively. The columns were preceded by an Oligopore 50 × 7.5 mm guard column. The volume of injection was 20 µL and detection occurred at 268 nm. GPC analysis was performed in isocratic mode employing dimethylformamide at a flow rate of 0.7 mL/min, at 70 °C. The sample was dissolved in the mobile phase solvent to obtain a concentration of 5 mg/mL, stirred until complete dissolution, and filtered through a 0.45 µm syringe filter before injection.

### 3.9. Solubility

The solubility of LCLig was determined using different solvents: deionized water, acetone, ethanol, methanol, and DMSO. The sample (10 mg) was suspended in 1 mL of solvent, and the mixture was homogenized and ultrasonicated for 15 min. The soluble and insoluble fractions were separated by centrifugation at 15,000 rpm (Megafuge 16R, Thermo Fisher Scientific); the insoluble fractions were oven-dried overnight at 105 °C (Venti-Line Model, VWR) and weighted for solubility determination. The experiments were carried out in triplicate.

### 3.10. Biological Activity

#### 3.10.1. Total Phenolic Content (TPC)

The TPC was evaluated using the Folin–Ciocalteu method, following the approach described by Vilas-Boas and co-workers [58]. In a 96-well microplate, 20 µL of sample or gallic acid or solvent (blank) were mixed with 80 µL of Folin–Ciocalteu reagent (10% *v*/*v*) and 100 µL of sodium carbonate (7.5 wt%). After allowing the reaction to proceed at room temperature for 1 h, the absorbance was measured at 750 nm using a microplate spectrophotometer (Epoch, Agilent, Santa Clara, California, USA). Triplicate analyses were carried out for each sample, and the results are expressed as milligram gallic acid equivalents (GAE) per gram of sample.

#### 3.10.2. Antioxidant Activity

The antioxidant activity was assessed by the oxygen radical absorbance capacity (ORAC) and 2,2′-azino-bis(3-ethylbenzothiazoline-6-sulfonic acid) (ABTS) radical cation assays. The lignin samples were prepared as described by Antunes and co-workers [35]. 

The ORAC assay was performed as described in the literature with some modifications [59]. Fluorescein and 2,2′-azobis(2-amidi-nopropane) dihydrochloride (AAPH) were used as fluorescent probe and peroxyl radical generator, respectively, and the reaction was conducted at 37 °C using a black polystyrene 96-well microplate (Nunc, Thermo Fisher Scientific, Waltham, CA, USA). Each well contained 200 mL of phosphate buffer (75 mM and pH 7.4), 20 µL of blank, sample or Trolox (1–8 µM), and 120 µL of fluorescein (70 nM). Prior to the addition of AAPH (60 µL; 12 mM), the mixture was preincubated for 10 min. Fluorescence measurements were recorded at 1 min intervals for a total duration of 140 min, using an excitation wavelength of 485 nm with emission detected at 530 nm (Synergy H1, Biotek Instruments, Winooski, Vermont, USA). The microplate was automatically shaken before each reading. This assay was performed in triplicate for each sample and the results are expressed as micromole Trolox equivalents (TE) per gram of sample. 

The ABTS assay was conducted including adjustments from established procedures [60]. The initial ABTS radical concentration was adjusted to achieve an absorbance of 0.700 (±0.020) at 734 nm. The reaction mixture consisted of 200 µL of ABTS radical and 15 µL of sample or butylated hydroxytoluene (BHT, reference antioxidant) or solvent (blank). After a 5 min incubation at 30 °C, the absorbance was measured in a microplate spectrophotometer at 734 nm. The experiments were performed in triplicate and the results are expressed as half-maximal inhibitory concentration (IC_50_), which represents the concentration of the sample required to scavenge 50% of the ABTS radicals.

### 3.11. Emulsion Stability Index (ESI)

To evaluate the ESI, the volumetric method proposed by Choi and co-workers was adopted [61]. The freshly prepared emulsion was poured into a glass volumetric cylinder and allowed to stand at 25 °C. This formulation was composed of 25 wt% cetyl stearyl alcohol (Lanette^®^ O, BASF, Ludwigshafen, Germany), 1–10 wt% of lignin, and 65–75 wt%. of water. The emulsion stability was recorded visually by the formation of the clarified serum layer at the bottom of the emulsion after 24 h. Then, the *ESI* was calculated as follows (1):

(1) $$ESI=\left(1-\frac{Vw}{Ve}\right)\times 100\;$$

where *Ve* is the volume of the o/w emulsion and *Vw* is the volume of the separated bottom layer after the desired storage period.

### 3.12. Safety Assessment

#### 3.12.1. Cytotoxicity

The cytotoxicity of LCLig on HaCAT cells was evaluated using the PrestoBlue Fluorescence assay (Thermo Fisher Scientific 2010). The cells were seeded, at 1 × 10^5^ cells/mL, in 96-well plates. Following a 24 h incubation at 37 °C in a humidified atmosphere of 5% CO_2_, the cells were exposed to the LCLig at concentrations ranging from 62.5 to 2000 µg/mL prepared in Dulbecco’s Modified Eagle Medium (DMEM). Cells treated with 10% DMSO were used as control. After a 24 h exposure, the cells were incubated with PrestoBlue reagent (Thermo Fisher Scientific). Cell viability was assessed through fluorescence spectroscopy after a 2 h incubation at 37 °C, using a Synergy HT Multi-detection microplate reader operated by GEN5TM software, version 2.0, Biotek, Instruments, Winooski, Vermont, USA, with excitation and emission wavelengths of 560 and 590 nm, respectively. The results are expressed as percentage relative to the untreated control cells (1% DMSO), considering the solvent control as 100% of viability.

#### 3.12.2. Mutagenicity

Three standard strains of *Salmonella typhimurium* were used to assess the mutagenic potential by the Miniaturized Ames Test in a liquid 384-well microplate format (Moltox^®^). Each strain contains a deletion mutation for histidine (His), with the following mutations: frameshift (TA98), base-pair substitution (TA100), and reversion/transversion (TA102). The strains were inoculated in nutrient broth-2 with 25 μg/mL of ampicillin and incubated for 10–12 h at 37 °C in an incubator shaker (Innova 44, New Brunswick) at 120 rpm until a density of 1–2 × 10^9^ bacteria/mL or approximately 1.0–1.4, OD 650 nm. Both assays were performed in the presence and absence of rat liver homogenate (S9) (30% *v*/*v*). A total of five LCLig concentrations were prepared in DMSO and added to the exposure medium (inoculum at 10% *v*/*v*). DMSO was used as negative control, and 2-nitrofluorene (100 µg/mL), 4-nitroquinoline-*N*-oxid, and mitomycin-C (50 µg/mL each) were used as positive controls for the strains TA98, 100, and 102, respectively. An aliquot of 2-aminoantracene (100 µg/mL) was used as a positive control for S9. The conesiversion indicator media was added after a 90 min incubation. The resulting mixture (50 μL) was moved to a 384-well plate, sealed into a plastic bag, and incubated at 37 °C for approximately 24 h for TA102 and 48 h for the other strains. After the incubation times, the growth of *Salmonella* his+ revertants was counted.

#### 3.12.3. Skin Sensitization

The skin sensitization potential was assessed through Direct Reactivity Peptide Assay (DPRA), following OECD Test guideline No. 442C (2020). LCLig was dissolved in acetonitrile:DMSO (2.3:0.7, *v*/*v*) at a concentration of 8 g/L, and further diluted to 1.4, 0.70, and 0.35 g/L. The peptides cysteine (0.667 mM stock solution in 100 mM phosphate buffer, pH 7.5) and lysine (100 mM stock solution in ammonium acetate buffer, pH 10.2) were used in the experiment. Cinnamic aldehyde (100 mM in acetonitrile) was used as positive control. The diluted samples were subjected to a 24 h incubation at room temperature, with cysteine or lysine peptides at ratios of 1:10 or 1:50 (*v*/*v*), respectively. The analysis was carried out using a high-performance liquid chromatography reverse-phase chromatograph (HPLC, Agilent 1260 Infinity II, Agilent Technologies, Santa Clara, California, USA) equipped with a diode array detector (Agilent 1260 DAD HS) and a Poroshell 120 EC C18 column (30 × 150 mm; 2.7 µm), in gradient mode with mobile phase A (0.1% trifluoroacetic acid in water) and phase B (0.085% trifluoroacetic acid in acetonitrile) starting at time 0 with 10% B, going to 25% B in 15 min, 90% B in 3 min, 10% B in 1 min and kept 6 min at 10% B. Detection was conducted at 220 nm, with a flow rate of 0.35 mL/min, column temperature of 30 °C, and a sample injection volume of 5 μL. Peptide quantification was achieved using calibration curves for cysteine and lysine in the range of 0.033 to 0.528 mM. The overall peptide depletion was determined considering the average % of cysteine and lysine depletion.

### 3.13. Accelerated Stability

The accelerated stability evaluation was performed according to the International Conference of Harmonization (ICH) guidelines ISO/TR 18811:2018 [62], for 3 months at different temperatures/related humidities: 4 °C/60%; 25 °C/60%; and 40 °C/75% (Climacell Eco line). The organoleptic (appearance, color, and odor) and physico-chemical (color, pH, and viscosity) parameters, ABTS radical scavenging capacity, and microbial contamination (ISO 11930:2019/ISO 17516) were monitored [63,64].

#### 3.13.1. Oil-In-Water (o/w) Emulsion Preparation

The oil-in-water (o/w) emulsion composition is detailed in Table 9. It was prepared by incorporating the oil phase into the water phase under high-shear mixing using an Ultra Turrax homogenizer (Ika^®^ T25, Staufen, Germany). In each phase, all ingredients were heated to 75 °C and blended vigorously. Aqueous phase (Part A) and oily phase (Part B) were combined. After cooling down, Euxyl PE 9010 (Part C) was added to the emulsion. The pH was corrected to 4.5–5.5 with sodium hydroxide 50 wt% solution or lactic acid.

#### 3.13.2. Organoleptic Evaluation

The appearance of the emulsions was visually monitored for indications of phase separation without reversion, creaming, or flocculation events. The color was evaluated according to the procedure mentioned above (Section 2.4).

#### 3.13.3. Physico-Chemical Evaluation

The pH was determined at 25 °C using a pH meter (Mettler Toledo, Greifensee · Switzerland), immersing the glass electrode into the emulsions until stabilization. The viscosity was measured using a Viscometer B-One Plus (Lamy Rheology Instruments, Champagne au Mont d’Or, France) with a spindle number R5 at 100 rpm for 60 s. All the measurements were performed at 25 °C with prior equipment calibration.

#### 3.13.4. Antioxidant Activity

The antioxidant activity was assessed following the abovementioned (Section 3.10.2) methodology with different sample preparation. The o/w emulsion (20 mg) was dissolved in 1 mL of DMSO and centrifuged at 5000 rpm for 30 s after ultrasonication for 15 min.

#### 3.13.5. Microbial Contamination

The microbial contamination was performed according to the European Standard ISO 17516:2014 (“International Organization for Standardization (ISO) 17516:2014—Microbiological Limits for Cosmetics” 2014) regarding total counts of yeast and mold, and total viable aerobic count (CFU/g) [64].

## 4. Conclusions

In this study, a simple process was successfully applied to reduce the color of lignin extracted from SCB. A detailed characterization of the resulting LCLig was performed, consisting of 81.6% total lignin, 3.5% carbohydrates, and 3.6% inorganics. LCLig showed a comparable molecular weight to previous SCB-derived lignins, indicating that the hydrogen peroxide treatment did not cause significant fractionation or degradation of the lignin structure. The analysis of the LCLig structure revealed reduced total phenolic units and carboxylic acids compared to the original SCB lignin, supporting the observed reduction in TPC and antioxidant activity. Furthermore, the color reduction treatment positively influenced the water solubility of LCLig. The obtained LCLig was found to be safe for topical application based on the results of cytotoxicity, mutagenicity, and skin sensitization tests. In addition, it was shown to effectively stabilize o/w emulsions at a minimum concentration of 10 wt% or 2.5 g per g of oil phase. Furthermore, its high ESI at lower concentrations (1.0%) suggests LCLig as a potential co-emulsifier, thus reducing the concentration of synthetic emulsifiers in cosmetic formulations. This is particularly relevant in the development of sustainable and more eco-friendly cosmetic formulations, as they rely less on synthetic emulsifiers while maintaining good emulsification, stability, and performance. All these findings support the conclusion that LCLig is a promising candidate for cosmetic applications.

## Figures and Tables

**Table 1:** Composition of light-colored lignin (LCLig), color and appearance.

Total Lignin (wt%)	Carbohydrates (wt%)	Ash (wt%)	Color CIELAB L*/a*/b*	Appearance
81.60 ± 3.60	3.50 ± 0.40	6.03 ± 0.01	73/2.5/24.5	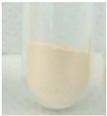

**Table 2:** Assignments and quantification of aliphatic, carboxylic OH groups, and phenolic OH groups identified by ^31^P NMR in light-colored lignin (LCLig).

Aliphatic OH (mmol/g _lignin_)	Carboxylic Acids (mmol/g _lignin_)	Phenolic Units (mmol/g _lignin_)				Total
		Condensed	Non-Condensed			
			S	G	H	
δ 146.4–150.8 mg/L	δ 135.6–133.6 mg/L	δ 145.8–143.8 and 142.2–140.2 mg/L	δ 143.8–142.2 mg/L	δ 140.2–137.4 mg/L	δ 137.4–136.9 mg/L	
3.73	1.76	0.13	0.10	0.63	0.04	0.89

**Table 3:** Results of the DSC analysis of light-colored lignin (LCLig).

Sample	Peak Max (°C)	Enthalpy (J/g)	Peak Height (mW/mg)
LCLig	74.9	−65.76	−0.6585
	159.2	−8.367	−8.367

**Table 4:** Particle size, molecular-average (Mw) and number-average (Mn) weights, and polydispersity index of the light-colored lignin (LCLig).

Sample	Particle Size (μm)					Mw (g/mol)	Mn (g/mol)	PD
	D[4,5]	D[3,4]	Dv(10)	Dv(50)	Dv(90)			
LCLig	13.88 ± 0.15	6.00 ± 0.02	2.83 ± 0.01	8.07 ± 0.02	27.24 ± 0.32	14,300 ± 2059	10,598 ± 1577	1.35

D[4,5] is the volume-based mean diameter and D[3,4] is the surface-area-based mean diameter. Dv(50), Dv(10), and Dv(90) are standard percentile readings expressed in volume distribution, where Dv(50) is the size in microns at which 50% of the sample is smaller and 50% is larger. This value is also known as the Mass Median Diameter (MMD) or the median of the volume distribution. Dv(10) is the size of particle below which 10% of the sample lies and Dv(90) is the size of particle below which 90% of the sample lies.

**Table 5:** Total phenolic content (TPC), oxygen radical absorbance capacity (ORAC), and 2,2′-azinobis [3-ethylbenzothiazoline-6-sulfonic acid]-diammonium salt (ABTS) IC_50_ of the light-colored lignin (LCLig).

Lignin	TPC (mg GAE/g)	ORAC (µmol TE/g)	ABTS * IC_50_ (mg/mL)
LCLig	169.3 ± 40.9	2571.5 ± 826	1.28 ± 0.13

GAE—Gallic acid equivalents; TE—Trolox equivalents; IC_50_—half maximal inhibitory concentration. * IC_50_ for butylated hydroxytoluene (BHT) was 0.40 ± 0.02 mg/mL.

**Table 6:** Emulsion stability index (*ESI*) for different amounts of light-colored lignin (LCLig).

LCLig (wt%)	*ESI* (%)
1.0	57.39 ± 1.33
2.5	65.49 ± 0.04
5.0	79.59 ± 2.56
7.5	85.52 ± 6.34
10.0	100.00 ± 0.00

**Table 7:** Cysteine (Cys) and lysine (Lys) depletion (%), reactivity, and prediction models for cinnamaldehyde (positive control) and sugarcane bagasse (SCB) light-colored lignin (LCLig).

Sample	Conc. (mg/mL)	Cys % Depletion	Cys and Lys % Depletion	Reactivity (Cys)	Reactivity Class	DPRA Prediction
Cinnamaldehyde (Positive control)	13.2	76 ± 3	66 ± 1	Moderate	High reactivity	Sensitizer
LCLig	1.40	6.0 ± 0.8	3.4 ± 0.4	Minimal	Minimal	No Sensitizer
	0.75	5.7 ± 0.6	2.8 ± 0.3			
	0.35	5.9 ± 0.4	2.8 ± 0.2			

Reactivity to Cys: Mean % Depletion ≤ 13.89% (No or minimal reactivity); Positive: 13.89% ≤ Mean % Depletion ≤ 23.09% (low reactivity); 23.09% ≤ Mean % Depletion ≤ 98.24% (moderate reactivity); Mean % Depletion ≥ 98.24 (high reactivity). Reactivity to Cys + Lys: 0% ≤ Mean % Depletion ≤ 6.38% (No or minimal reactivity); Positive: 6.38% ≤ Mean % Depletion ≤ 22.62% (low reactivity); 22.62% ≤ Mean % Depletion ≤ 42.47% (moderate reactivity); 42.47% ≤ Mean % Depletion ≤ 100% (high reactivity).

**Table 8:** Stability results for blank and 5 wt% light-colored lignin (LCLig) o/w emulsions across 3 months under different conditions (4, 25, and 40 °C).

Parameter	T (°C)	Blank o/w Emulsion				5 wt% LCLig o/w Emulsion			
		Initial	1 Month	2 Months	3 Months	Initial	1 Month	2 Months	3 Months
Physical appearance	4	Homogeneous and smooth consistency; good spreadability				Homogeneous and smooth consistency			
	25								
	40								
Color L* a* b*	4	90.42 −0.68 2.88	81.65	82.92	87.43	55.1 7.4 25.7	53.00	52.54	53.13
			−0.84	−0.37	−0.68		5.70	5.38	7.19
			3.32	2.00	2.46		20.46	23.29	23.48
	25		83.53	84.99	86.8		52.54	52.60	52.74
			−0.96	−0.48	1–0.6		5.38	7.02	6.76
			4.05	2.85	3.51		20.38	23.29	21.47
	40		81.87	84.43	88.07		51.74	52.13	52.77
			−0.83	−0.53	−0.97		5.33	6.88	6.84
			3.68	2.89	3.42		19.66	21.28	20.87
pH	4	5.33 ± 0.02	3.89 ± 0.02	4.13 ± 0.02	4.40 ± 0.02	5.4 ± 0.07	5.34 ± 0.07	5.26 ± 0.07	5.41 ± 0.07
	25		3.74 ± 0.02	3.87 ± 0.02	4.28 ± 0.02		5.31 ± 0.07	5.26 ± 0.07	5.33 ± 0.07
	40		3.73 ± 0.02	3.87 ± 0.02	4.59 ± 0.02		5.34 ± 0.07	5.27 ± 0.07	5.43 ± 0.07
Viscosity (mPa s)	4	1561 ± 8	1957 ± 93	1961 ± 83	1628 ± 54	1414 ± 17	1507 ±1	1870 ± 14	1491 ± 13
	25		1991 ± 95	1968 ± 43	1883 ± 06		1768 ±1	1730 ± 2	1815 ± 4
	40		1715 ± 127	1961 ± 83	1628 ± 54		1507 ± 1	1870 ± 14	1491 ± 13
Antioxidant activity ABTS, IC50 (mg/mL)	4	Not detected				0.64 ± 0.07	0.91 ± 0.03	0.91 ± 0.15	0.67 ± 0.03
	25						0.89 ± 0.05	0.83 ± 0.05	0.73 ± 0.09
	40						0.93 ± 0.04	0.55 ± 0.04	0.60 ± 0.03
Total counts of yeast and mold (CFU/g)	4	Absent				<10			
	25								
	40								
Total viable aerobic count (CFU/g)	4	<10				<10			
	25								
	40								

**Table 9:** Composition of light-colored lignin (LCLig) and blank emulsions.

Commercial Name	INCI	Function	Blank (%)	LCLig (%)
Part A (aqueous phase)				
Deionized water	Aqua	Solvent	79.6	74.6
Glycerin	Glycerin	Humectant	5	5
SolagumTM AX	Acacia Senegal Gum; Xanthan Gum	Thickening and stabilizing agent	0.9	0.9
LCLig	-	Active ingredient	-	5
Part B (oily phase)				
Lanette	Cetyl stearyl alcohol	Emulsifier	2.5	2.5
Tego^®^ Care PBS 6 MB	Polyglyceryl-6 Distearate; Polyglyceryl-6 Behenate	Emulsifier	4	4
Shea butter	Butyrospermum parkii butter	Emollient	2	2
Squalane	Squalane	Emollient	5	5
Part C				
Euxyl PE 9010	Phenoxyethanol and Ethylhexylglycerin	Preservative	1	1
